# Potential of innovations in hygiene management – a managerial perspective

**DOI:** 10.1186/s13756-019-0555-x

**Published:** 2019-06-14

**Authors:** Luise Hutzschenreuter, Nils-Olaf Hübner, Kathleen Dittmann, Angela-Verena Hassel, Steffen Flessa

**Affiliations:** 1 Department of Health Care Management, University of Greifswald, Friedrich-Loeffler-Str. 70, 17487 Greifswald, Germany; 2 Department of Infection Prevention and Control and Institute for Hygiene and Environmental Medicine, University Medicine of Greifswald, Walther-Rathenau-Straße 49a, 17489 Greifswald, Germany

**Keywords:** AHOI–Patients on board, Infection prevention, Patient safety, Hygiene management, Infection control, Innovation, Patient involvement

## Abstract

**Background:**

Assessment of the current situation is crucial before introducing innovative infection prevention measures. According to the literature, hospital managers should take on the role of “power promoters” in adopting infection prevention measures due to their position and decision-making authority. However, there is no empirical evidence for whether or not this assumption is valid. This paper reports German hospital managers’ perceptions of current challenges in infection prevention and control and innovative prevention measures. We analysed the managerial promoters and barriers of adopting innovations in order to derive recommendations for improving the innovation process in hospitals using the novel AHOI-approach to actively involve patients and their relatives in anti-infection measures.

**Methods:**

All 3877 medical, nursing and administrative managers of German hospitals were invited to participate in an online survey. The first set of questions intended to determine their perception of problems of hygiene management in their institution and in particular in the interaction with patients and their relatives. The second set of questions was asked to identify potential challenges and barriers to combating nosocomial infections and involving patients and their relatives in infection prevention.

**Results:**

Two hundred six managers from German hospitals participated in the survey. Transmission of pathogens was seen as the main problem in the inpatient area, especially in acute care hospitals and stationary geriatric care. Barriers to the implementation of novel infection prevention concepts were primarily perceived as lack of time and refinancing by health insurance providers. The surveyed hospital managers assessed that the active involvement of patients and their relatives in infection prevention could strengthen the infection prevention of their institution.

**Conclusions:**

Hospital managers are open to innovative hygiene interventions. In particular, they welcome the active involvement of patients and their relatives in infection prevention. Therefore, financial and institutional barriers, such as insufficient funding of hygiene management, must be overcome.

## Introduction

Hygiene and infection control are essential elements of a hospital’s quality management system and must be kept up to date, which requires constant innovation [1–3]. According to the promoter model of the theory of innovation, at least two different key persons must exist to advance and implement innovations in an organization [4]: the professional promoter, who overcomes the barrier of “not knowing” the innovative approach, and the power promoter who helps to overcome the barrier of “not wanting” within the organization [5, 6]. While infection prevention and control specialists serve as professional promoters, hospital managers are supposed to serve as power promotors.

In Germany, hospital managers are legally responsible for the design and implementation of infection prevention measures (§ 135a German Social Code (SGB) V, § 23 Infection Control Act (IfSG)) as part of the quality management system [7, 8]. Therefore, the medical, nursing and administrative managers are natural members of the hospital’s hygiene board to promote up-to date infection improvement measures [1, 9].

As power promoters, hospital managers must analyse the existing situation, compare it with their institutional goal system, analyse pros and cons of alternative solutions, and enforce the adoption of innovations if they realize that the innovative approach produces better results, making the investment worthwhile.

However, little is known about the perceived relevance of nosocomial epidemiology and perceived barriers to implementing infection prevention and control measures by hospital managers. This applies to measures of the standard model of infection prevention, which mainly focusses on changing the behaviour of medical and nursing staff to prevent nosocomial infections, it applies even more for the novel concept of actively involving patients and their relatives in the prevention of infection in healthcare institutions [10, 11].

Therefore, the aim of this study was to close this research gap by evaluating the assessment of the hygiene situation in German hospitals and the approach of active involvement of patients and their relatives in hospital hygiene by German hospital managers. The results were used to analyse and assess the status quo of infection prevention strategies and related healthcare challenges from the perspective of the power promoters, who play a key role in the adoption of innovative prevention approaches.

## Methods

Our approach is based on the assumption that innovations in hospitals, especially in hygiene management, depend on fulfilling three requirements. First, leaders need to identify the problem that the innovation is intended to overcome. Second, the willingness of leaders to take leadership in the innovation process must exist. Third, they must estimate the costs and compare them with the benefits of innovation [5]. The aim of this survey, which was part of the project “AHOI – Patients on Board” (Activation of patients, people in need of care and care-providers for a Hygiene-conscious participatiOn in Infection prevention), was to assess the perception of problems of hygiene management in German hospitals and in particular regarding the interaction with patients and their relatives. In addition, this study aimed to identify potential challenges and barriers to combating nosocomial infections and to the integration of patients and their relative in infection prevention. In the project “AHOI – Patients on Board”, patients and their relatives are systematically involved in infection prevention. This project is based at the Institute for Hygiene and Environmental Medicine of the University Medicine Greifswald.

### Questionnaire design

The development of the questionnaire followed a three step-approach: First, interviews with three key experts in the field of hygiene management were conducted. The interviews were based on a semistructured interview guide. Second, based on the interviews, a questionnaire was developed and afterwards tested and validated in nine further interviews with healthcare managers. Third, after completing the interviews and further developing the questionnaire, the questionnaire was finalized.

### Questionnaire structure

The questionnaire was divided into the following topics: 1) general information about the hospital and the interview partner, 2) experience in infection prevention and control management, 3) assessment of the hygiene situation, 4) importance of patients and their relatives for the hygiene process, and 5) efficiency, effectiveness and willingness to change the hygiene management. Each of these topics consisted of several items. The questionnaire contained only closed questions with nominal (e.g., “yes” or “no”) and ordinal Likert scales (four-level, five-level and ten-level rating scales). For one question, multiple answers were possible, otherwise only one answer was allowed.

### Conducting the survey

The aim was to give all German hospital managers (medical, administrative and nursing manager) the opportunity to participate in this survey. For this purpose, an online survey with the evaluation and survey software EvaSys (Electric Paper Evaluationssysteme GmbH), under the premise of effectiveness and conservation of resources, was chosen as the survey tool. The invitation to participate in the survey and the link to the online survey were sent to all e-mail addresses of German hospital administrators available at www.german-hospital-directory.com (status as of June 2018). As assured in the cover letter, participation was anonymous and voluntary. The survey period was 6 weeks, from 25 July 2018 to 5 September 2018.

### Data analysis

The statistics software IBM SPSS Statistics 25 and MS Office Excel 2016 were used for statistical analysis. Methods of descriptive statistics such as mean value (MV), standard deviation (SD) and 95% confidence interval (CI) helped to describe the data. In addition, correlation analyses, depending on the scale level of variables, were performed, such as Cramer’s V and Spearman’s rank correlation. Furthermore, nonparametric tests (Kruskal-Wallis and Dunn-Bonferroni’s post hoc tests) were carried out. A significance level of 5% (*p* = 0.05) was assumed for all data analyses in this study.

## Results

### Response rate and sample description

Of the 3877 hospital managers asked to participate, 206 completed the online survey. This corresponds to a return rate of 5.3%. The sample consisted of 124 men (63.3%) and 71 women (36.7%). The age structure of the respondents is shown in Table 1. Eighty-three participants (42.2%) were between the ages of 56 and 65. The second-largest age group, with 77 participates (39.8%), was 46- to 55-year-olds.

**Table 1:** Age structure of participating hospital managers separated by sex (missing cases: *n* = 11)

Age group	Female		Male	
	N	%	n	%
≤35 years	1	1.4%	2	1.6%
36 to 45 years	14	19.7%	15	12.1%
46 to 55 years	33	46.5%	44	35.5%
56 to 65 years	22	31.0%	61	49.2%
> 65 years	1	1.4%	2	1.6%
Total	71	100%	124	100%

Broken down by profession, 75 (36.4%) medical managers, 91 (44.2%) nursing managers and 29 (14.1%) administrative managers participated in the study. Eleven (5.3%) respondents did not state their position at the hospital. The distribution of the sample by numbers of beds shows that the largest group of managers (*n* = 82; 39.8%) led a hospital between 150 and 399 beds (Table 2).

**Table 2:** Structure of the sample by hospital size (numbers of beds)

Numbers of beds	N	%
< 50	8	3.9%
50 to under 150	52	25.2%
150 to under 400	82	39.8%
400 to under 650	40	19.4%
650 and more	24	11.7%
total	206	100%

### Responsibility of hygiene management

One hundred thirty-four (65.0%) managers were responsible for the hygiene management of their hospital. Seventy-one of 75 (94.7%) medical managers reported being responsible for the hygiene management of their institution. Thirty-eight of 91 (41.8%) nursing managers and 16 of 29 (55.2%) administrative managers reported being responsible for the hygiene management of their institution. Based on a scale of ten, participants were asked to assess their own level of knowledge in hygiene management (from 1 “no knowledge” to 10 “extensive knowledge”). On average, medical managers rated their knowledge of hygiene management as highest (MV = 7.99, 95% CI 7.60–8.37), followed by nursing managers (MV = 7.90, 95% CI 7.52–8.28) and administrative managers (MV = 7.28, 95% CI 6.52–8.04).

### Participation in hygiene training

**Fig. 1:**
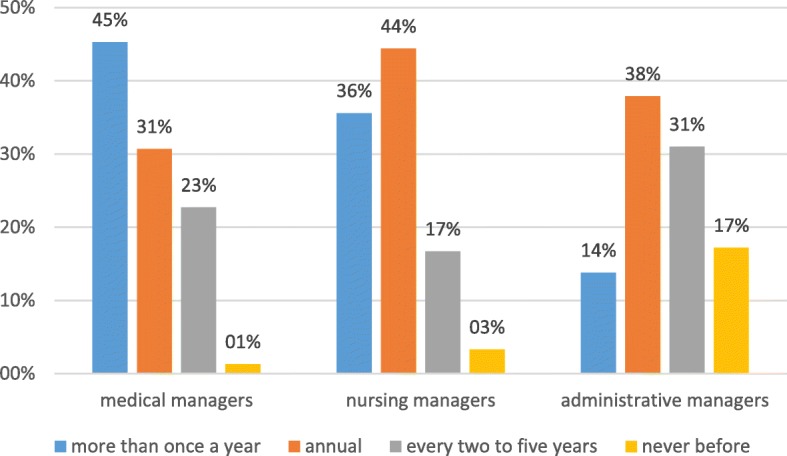
Participation frequency in hygiene training broken down by occupational category (*n* = 194)

Regarding participation in hygiene training, 70 (36.1%) hospital managers indicated that they attend more than once a year. Seventy-four (38.1%) respondents participated annually, 41 (21.1%) participated every two to five years and 9 (4.6%) did not participate in any hygiene training. The frequency of participation in hygiene trainings by managerial position shows that almost half of the administrative management (14/29 participants) said they never or rarely attended hygiene training (Fig. 1). Fifty-seven of 75 (76%) of the medical managers said that they attended hygiene training at least once a year. Seventy-two of 90 (80%) nursing managers stated that they attended hygiene training at least once a year. Twelve (5.8%) participants did not provide information.

### Assessment of the hygiene situation

**Fig. 2:**
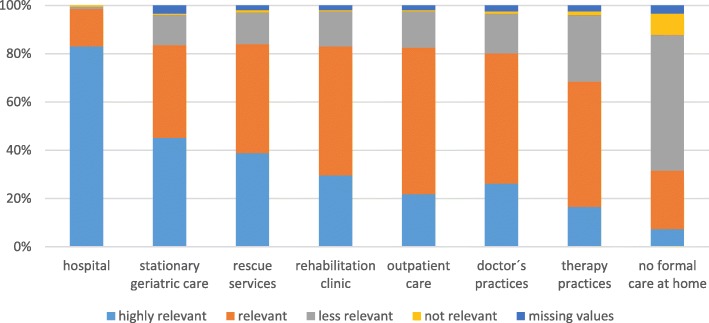
Managers’ assessment of the relevance of the pathogen transmission problem in different areas of patient care

Hospital administrators were asked to assess the relevance of the problem of pathogen transmission in different areas of the German health care system (Fig. 2). In the respondents’ opinion, the problem of inpatient transmission (MV = 3.4) is more relevant than in the outpatient sector (MV = 2.9). The highest relevance was attributed to in the hospital (MV = 3.8, SD = 0.5), followed by stationary geriatric care (MV = 3.3, SD = 0.7) and rescue service (M = 3.2, SD = 0.7), rehabilitation clinics (MV = 3.1, SD = 0.7), doctor’s practices (MV = 3.1; SD = 0.7), outpatient care (MV = 3.1, SD = 0.6) and in therapy practices (MV = 2.9, SD = 0.7). The problem of pathogen transmission through no formal care at home was considered less relevant than the other areas (MV = 2.3, SD = 0.7).

According to the breakdown in the hospital, the problem of pathogen transmission both in intensive care units (MV = 3.9, SD = 0.2) and operation theater units (MV = 3.8, SD = 0.5) was considered highly relevant, whereas in non-operative units (MV = 3.5, SD = 0.5) it was considered relevant.

The assumed effectiveness of measures to prevent pathogen transmission was assessed based on the 4-step scale: 1 = “very ineffective”, 2 = “rather ineffective”, 3 = “rather effective” and 4 = “very effective”. Table 3 shows the results of the analysis. The presence of hygiene personnel (MV = 3.8, SD = 0.4), regular compulsory hygiene training of personnel with a focus on hand hygiene (MV = 3.8, SD = 0.5), inpatient screening in risk groups (MV = 3.6, SD = 0.6) and reprocessing of medical devices (MV = 3.6, SD = 0.6) were evaluated as very effective prevention measures. Outpatient screening for multidrug-resistant organisms (MDRO) (MV = 3.4; SD = 0.7), active involvement of patients and their relatives in infection prevention (MV = 3.3, SD = 0.7) and cooperation with research institutions (e.g., quantifying disinfectant consumption) (MV = 3.1, SD = 0.8) were considered to be rather effective measures to prevent pathogen transmission. The analysis of the relationship between the assessment of measures to prevent the pathogen transmission and profession showed a low correlation between the respondent’s profession and evaluation of “cooperation with research institutions” (Cramer’s V = 0.214), which was significant (*p* = 0.01). There was also a low correlation between the assessment of “active involvement of patients and their relatives in infection prevention” and the profession of the interview partner (Cramer’s V = 0.199), which was significant (*p* = 0.02). The correlation between the assessment of t “hygiene training of personnel” and profession was also low (Cramer’s V = 0.197), which was significant (*p* = 0.02).

**Table 3:** Analysis of the correlation between the evaluation of measures to prevent pathogen transmission and the profession of the interview partner

Measures to prevent pathogen transmission	Medical managers (MV)	Nursing managers (MV)	Administrative managers (MV)	Cramer’s V	*p*-value
cooperation with research institutions	3.0	3.2	2.9	0.214	0.01*
reprocessing of medical devices	3.5	3.7	3.7	0.202	0.02*
active involvement of patients and their relatives in infection prevention	3.2	3.5	3.1	0.199	0.02*
hygiene training of personnel	3.7	3.8	3.8	0.197	0.02*
Outpatient MDRO screening	3.2	3.5	3.4	0.157	0.15
Inpatient MDRO screening in risk groups	3.6	3.6	3.7	0.105	0.64
hygiene personnel	3.8	3.8	3.8	0.099	0.70

* significant correlation

### Barriers to the implementation of infection control measures

**Fig. 3:**
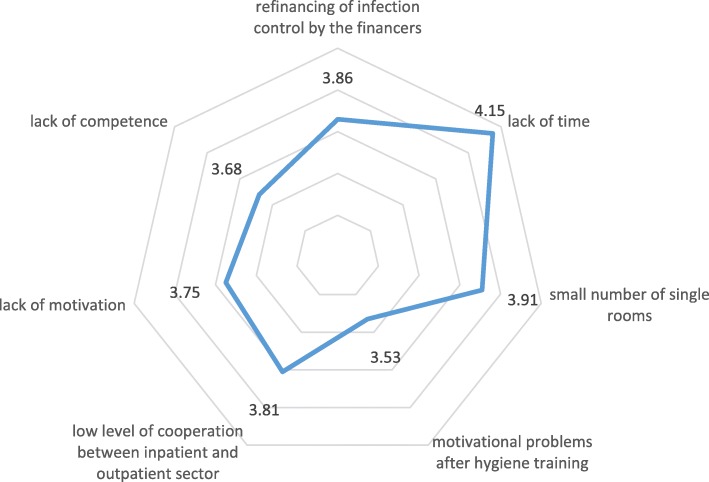
Assessment of the barriers to the implementation of infection control measures (1 “no barrier” to 5 “very strong barrier”)

The participants identified a number of factors which they felt hindered the implementation of infection control measures (Fig. 3). Mean value comparisons of the items based on the 5-level unipolar Likert scale (“no barrier” to “very strong barrier”) showed the greatest barrier to implementing infection prevention measures was lack of time on the part of the staff (MV = 4.1, SD = 0.9). The next most frequently identified barriers were small numbers of single rooms (e.g., necessary to isolate patients with multidrug-resistant pathogens, MV = 3.9, SD = 0.9) and the refinancing of infection control concepts by the financers of the German health care system (MV = 3.9, SD = 1). The latter comprise the statutory and private health insurance providers as well as the employers’ liability insurance association. A low level of cooperation between the outpatient and inpatient sectors, especially regarding the exchange of information, was seen as a rather strong barrier (MV = 3.8, SD = 0.9), followed by lack of motivation (MV = 3.7, SD = 1.1) and lack of competence of the staff (MV = 3.7, SD = 1.1). Motivational problems of the employees after hygiene training (MV = 3.5, SD = 0.9) was rated as the weakest barrier.

**Fig. 4:**
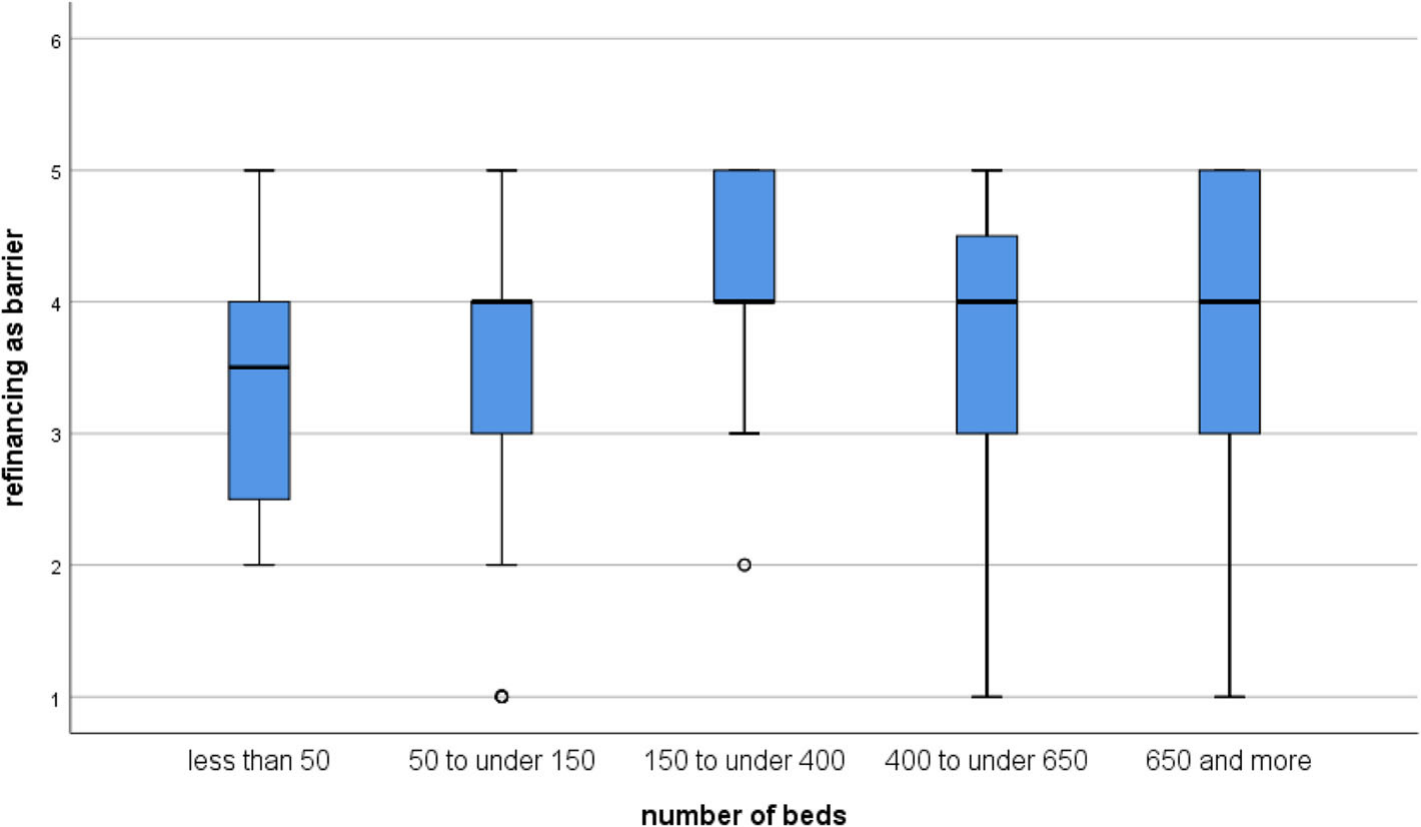
Assessment of the barrier refinancing (from 0 “no barrier” to 5 “very strong barrier”) according to hospital size (number of beds)

Depending on the hospital size (number of beds), the participants differed significantly in how they rated the refinancing of hygiene concepts as a barrier (Kruskal-Wallis test, *p* = 0.01). For hospitals with less than 50 beds, the refinancing of the hygiene management is a lower barrier than for hospitals with 50 and more beds (Fig. 4).

Medical (MV = 4.39) and nursing (MV = 4.04) managers rated the lack of resources as a larger barrier to the implementation of hygiene concepts than did administrative managers (MV = 3.76) did. Between nursing and administrative managers (*p* = 0.02, Dunn-Bonferroni test) as well as between administrative and medical managers (*p* = 0.002, Dunn-Bonferroni test), there were significant differences in the assessment of lack of time as a barrier. Between medical and nursing managers, there were no significant differences in the rating of this barrier (*p* = 0.76, Dunn-Bonferroni test).

### Importance of patients and their relatives for hygiene management

**Fig. 5:**
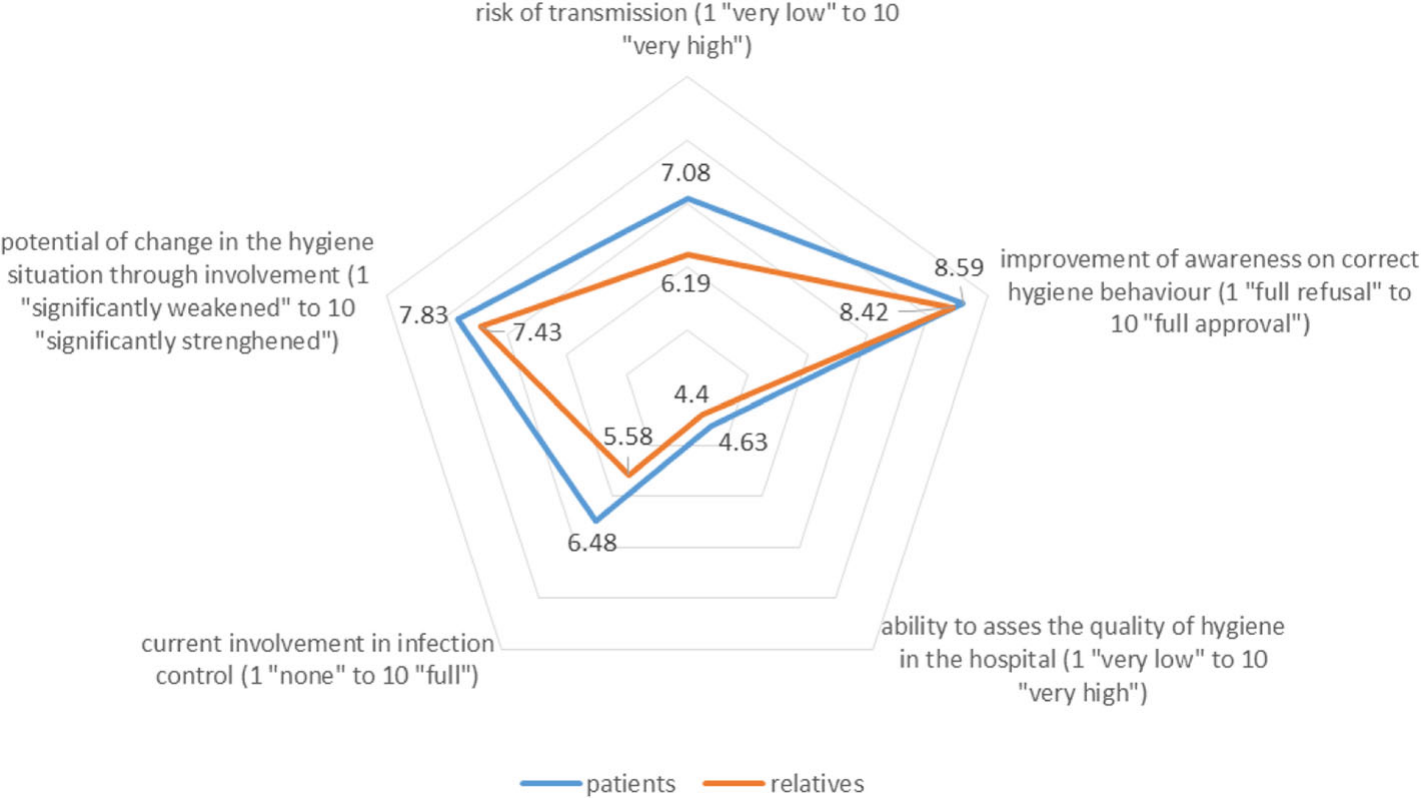
Assessment of respondents about the impact of patients and their relatives for the hygiene process

The surveyed managers estimated that the hygiene behaviour of patients poses a greater risk of transmission of pathogens (MV = 7.08, 95% CI: 6.75–7.41) than the behaviour of their relatives (MV = 6.19, 95% CI: 5.84–6.53) (Fig. 5). One hundred eighty-eight (91.2%) respondents stated that patient awareness of correct hygiene behaviour needed improvement (MV = 8.59, 95% CI: 8.34–8.83). Of the managers, 186 (90.7%; MV = 8.42, 95% CI: 8.15–8.68) saw the need to improve the knowledge of relatives about correct hygiene behaviour The ability of patients and their relatives to assess the quality of hygiene was rated as mediocre (patients: MV = 4.63, 95% CI: 4.35–4.91; relatives: MV = 4.40, 95% CI: 4.10–4.69). The different professional groups differed in their rating of patients’ ability to assess the quality of hygiene (medical managers: MW = 4.18, 95% CI: 3.75–4.6; nursing managers: MW = 5.05, 95% CI: 4.59–5.52; administrative managers: MW = 4.21, 95% CI: 3.67–4.74). Differences between the groups also existed for the assessment of the ability of relatives to judge hygiene quality (medical: MV = 3.86, 95% CI: 3.42–4.31; nursing: MV = 5.11, 95% CI: 4.65–5.57; administrative: MV = 3.48, 95% CI: 2.92–4.05).

At the time of the survey, in 9 (4.5%) surveyed institutions, patients were not included in infection control. In 34 (16.8%) institutions, patients were only slightly involved, in 120 (59.1%) hospitals patients were partially involved, and in 40 (19.7%) of the institutions, patients were very involved in infection control. Overall, patients (MV = 6.48, 95% CI: 6.17–6.80) were involved more often than relatives (MV = 5.56, 95% CI: 5.21–5.90) in infection prevention at hospitals. One hundred eighty-five (90.7%) managers indicated that integrating patients could strengthen hygiene control (MV = 7.83, 95% CI: 7.57–8.08). One hundred seventy (83.3%) respondents felt that involving patients’ relatives in hygiene management would improve the hygiene situation (MV = 7.43, 95% CI: 7.13–7.73).

Managers were asked for their opinions on how hospital staff reacted when patients or their relatives pointed out instances inadequate hygiene. On average, hospital managers estimated that their staff responded slightly negatively to such information from patients or their relatives (MV = 5.0, 95% CI: 4.64–5.35 on a scale from 1 = “negative” to 10 = “positive”).

### Effectiveness, efficiency and willingness to change

An important factor in the implementation and realisation of hygiene concepts is the financing of the planned measures. Twenty-seven (14.6%) respondents indicated that hygiene measures in their institution are sufficiently refinanced by health insurance providers. In 158 (85.4%) institutions, the hygiene measures were not adequately refinanced by health insurance providers. There were no significant correlations between the items occupational group (*p* = 0.82), number of beds (*p* = 0.10), medical care level (*p* = 0.49) or gender (*p* = 0.59) for the item “Are hygiene measures in your institution sufficiently refinanced by health insurance providers?”

The willingness of the hospital management to provide funds for the implementation of new infection prevention measures depends on various factors. One hundred thirty-six (65.7%) respondents would provide funding if the hygiene situation in their hospital stood to improve by implementing new infection control concepts. One hundred seventeen (56.8%) respondents would provide funding if the effectiveness of new infection prevention measures were already proven. One hundred twelve (54.1%) hospital administrations would provide funding if costs and benefits of the new infection prevention measures were known in advance. Potential improvement of the institution’s reputation by realising the new concept (decision criteria for 60 [29%] respondents) and the fact that implementation would include patients and relatives in infection prevention (decision criterion for 43 [20.8%] respondents) were less important decision-making factors in the provision of funding a new concept. There was a very low, non-significant negative correlation (*p* > 0.05) between the age of the managers and the level of their self-assessed innovativeness (r_s_ = − 0.029, Spearman rank correlation).

## Discussion

The surveyed hospital managers saw the problem of pathogen transmission mainly in the inpatient sector, especially in acute care hospitals and stationary geriatric care. Barriers to the implementation of hygiene concepts were primarily perceived as lack of time on the part of the staff and refinancing by health insurance providers. The surveyed hospital managers assessed that the active involvement of patients and their relatives in infection prevention could strengthen hygiene control at their institution.

### Infection prevention measures

The surveyed hospital managers evaluated the presence of hygiene personnel and regular hygiene training with a focus on hand hygiene as very effective for infection prevention. This reinforces the importance and need for hygiene personnel to prevent infection in the hospital. Today, these measures are standard in many hospitals. The German Hospital Structure Act (Krankenhausstrukturgesetz) strengthens the quality of hospital care and envisages targeted training and continuing education for hygiene professionals [12].

A large number of studies have proven the effectiveness of admission screenings in battling the transmission of MDROs [13–15]. In our study, MDRO screening in risk groups and (pre-admission) outpatient MDRO screening for planned hospital admissions were found to be effective measures to prevent transmission of pathogens. Data from the German National Reference Center for Surveillance of Nosocomial Infections (NRZ) showed that more than 90% of MRSA carriers are detected upon admission to German hospitals (reporting period: January until December 2017) [16]. This shows that many patients take these pathogens to the hospital. The idea to implement pre-admission MDRO screening for outpatient results from this finding. Initial studies for the feasibility of outpatient MDRO screening in Germany and the related costs have already been published [17, 18]. Nevertheless, reimbursement of this outpatient service by the health insurance providers is currently not available in Germany [19].

The active involvement of patients and their relatives in infection prevention has been proposed as an effective measure of preventing pathogen transmission. To date, however, this concept has scarcely been researched and must be seen as experimental. In the project “AHOI-Patients on Board”, this approach has been systematically developed based on and the three-pillars strategy “adherence”, “empowerment” and “acceptance”. In the first step, patients are provided with knowledge of hygiene standards and shown how to translate them into their own behaviour (adherence). In the second step, because the patients are aware of the hygiene measures the staff are required to perform, patients are able to give feedback to the staff and, if necessary, to demand compliance with the hygiene standards (empowerment). The third step is to enable the staff to stimulate, appreciate and improve the participation and feedback of the patients and their relatives (acceptance). The feasibility of the approach of “AHOI – Patients on board” to actively involve patients and their relatives in infection prevention has already been shown [11].

### Solutions to overcoming barriers

Lack of time on the part of the hospital staff and refinancing of hygiene measures by the health insurance providers were rated as the strongest barriers to implementing new hygiene concepts by the hospital managers. The first barrier shows that new concepts must not increase the workload of the staff, but must be integrated into the workflow in the best possible way. Since 2013, a special hygiene program for hospitals has existed in Germany, financed by the health insurance providers [20]. From 2013 to 2019, additional funds have been available to hospitals for recruiting, training and developing hygiene staff. At the same time, funds for external consulting services and undifferentiated funding for the prevention of nosocomial infections have been provided according to the recommendations of the Commission for Hospital Hygiene and Infection Prevention (KRINKO) [21]. Starting in 2020, this hospital-specific subsidy for the special hygiene program will result in permanent supplementary financing for all hospitals by including it in the base rate of the German Diagnosis-Related Groups (DRG) system. By the end of 2017, approximately 87% of the eligible hospitals had used funds from the special hygiene program [20]. This program together with the increase in the base rate by supplements for hygiene tasks represents a solution for overcoming the barrier of refinancing new infection prevention measures. The effects are not yet known.

### Importance of patients and relatives for the hygiene process

Little research has been done on the importance of patients and their relatives for the hygiene process in the hospital. Hygiene concepts that include an active role of patients in infection prevention require the support of the hospital management. Therefore, it is essential that hospital managers become acquainted with and evaluate the importance of patients and their relatives for hygiene process in order to build on this knowledge and develop appropriate preventive hygiene concepts.

The surveyed hospital managers evaluated the ability of patients and their relatives to assess the quality of hygiene as rather poor, and estimated that the knowledge of patients and their relatives about correct hygiene behaviour needs improvement. In order to enable patients to assess the quality of hygiene in the hospital, it is necessary to educate patients and their relatives about correct hygiene behaviour during their stay in the hospital. Our data show that respondents assessed that patients and their relatives would not or only partially be included currently in infection control. A large proportion of the interviewed hospital managers stated that the hospital’s hygiene situation could be strengthened by involving the patients and their relatives. This emphasizes the great potential for intensifying the infection prevention concepts by involving these relevant groups of persons. Nevertheless, the inclusion of staff should not be forgotten. It is important that training be provided especially on issues such as the recognition and promotion of the patient as a partner in infection prevention. “AHOI – Patients on board” has developed a variety of informative materials and intervention tools for explaining hygiene-relevant behavior to patients and staff. These materials consist of information brochures, various reminders and video presentations for patients and their relatives [11].

### Role of hospital managers as power promoters

Based on our survey, we can state that hospital managers are well informed about the state-of-the-art of infection prevention irrespective of their profession. Consequently, it is likely that they take their managerial and legal responsibility for hospital hygiene seriously. They also seem to critically examine innovations, such as patient participation, and seem to weigh the pros and cons of these innovative approaches. Based on their conclusion that a shortage of funding is a major cause for not implementing innovations, we can also state that hospital leaders analyze the cost-effectiveness of innovations, at least intuitively.

Consequently, managers of German hospitals are capable of and willing to take on the role of power promotors for hygiene innovations if the innovation pays off or if the health insurance providers refund the additional costs. For the first case, hospital managers need sufficient information on the costs and savings induced by innovative measures [22]. This is sometimes not easy to calculate. For instance, the costs of involving patients and relatives can be assessed (e.g., printing posters, producing videos, training and motivating staff), while the savings are difficult to predict (e.g., reduced infections, reduced lengths of hospital stay, improved demand for services due to better reputations). Theoretically, it is possible that the investment in this innovation pays off, but it would require intensive research to prove it.

If the additional costs of the innovative hygiene intervention concept do not pay off or savings are unknown, the additional costs of hygiene would have to be covered by the DRG system of the German health insurance providers. The DRG system is designed so that a hospital receives a certain amount for a patient irrespective of the investment in this patient, i.e., whether the patient receives an innovative hygiene intervention or not, the reimbursement will be the same [23]. Currently, a large number of German hospitals are not profitable and there is no potential for additional or increasing costs unless the health insurance providers refund these costs [24].

### Limitations

This study intended to provide a first assessment of hospital manager’s attitudes towards innovative infection prevention concepts, a perspective that has been scarcely researched. The relatively low response rate may indicate that managers are not used to be questioned about infection control measures or do not realise that they are part of their expertise or responsibility. Since the survey was based on voluntary participation, it can be assumed that especially persons who are responsible for hygiene management or who are interested in the subject of hospital hygiene were more likely to participate than those who were not. The results therefore suffer from a certain grade of selection bias. Further, the risk of social desirability bias should not be neglected, but the utmost care was taken in designing the questionnaire to maximize the anonymity of the participant and his/her hospital in order to minimize the risk of being susceptible to social desirability [25]. Likewise, the possibility of not having to answer certain questions existed. As the survey was anonymous, no statements about the large group of non-participant hospital managers can be made. While the sample is too small solidly represent the population, our results are still the best estimate in this field so far. Overall, the study meets the quality criteria of objectivity, reliability and validity of quantitative research.

## Conclusions

This study showed that new hygiene concepts are continuous challenges for hospitals and in particular for hospital managers. Regarding the problem of transmission of pathogens, hospital managers do not confine themselves to the hospital, but involve other participants in the health care system. Hospital managers are well informed about the state-of-the-art in infection prevention. To improve infection prevention and control, they are open to innovative hygiene interventions. In particular, they welcome active involvement of patients and their relatives. Financial and institutional barriers, such as insufficient funding of hygiene management and staff shortage in high-risk areas such as intensive care and surgery units, must be overcome. In conclusion, there is a necessity for comprehensive hygiene management that is constantly developing and open to innovative intervention concepts.

## Data Availability

The datasets used and/or analyzed during the current study are available from the corresponding author upon reasonable request.

## Abbreviations

CI: Confidence interval
DRG: Diagnosis Related Groups
IfSG: Infection Control Act
MDRO: Multidrug-resistant organisms
MV: Mean value
NRZ: German National Reference Center for Surveillance of Nosocomial Infections
SD: Standard deviation
SGB: German Social Code

## References

[1] KRINKO. Personelle und organisatorische Voraussetzungen zur Prävention nosokomialer Infektionen: Empfehlung der Kommission für Krankenhaushygiene und Infektionsprävention. Bundesgesundheitsbl. 2009(52):951–62. 10.1007/s00103-009-0929-y 19690813

[2] Rüden H, Gastmeier P, Daschner F (2000). Krankenhausinfektionen: Empfehlungen für das Hygienemanagement.

[3] Weigert J. Hygienemanagement und Infektionsprophylaxe: Ein praktischer Leitfaden für teil- und vollstationäre Pflegeeinrichtungen. 1st ed. s.l.: Schlütersche; 2010. 320 p. ger.

[4] Witte E. Organisation für Innovationsentscheidungen: Das Promotoren-Modell. Schriften der Kommission für Wirtschaftlichen und Sozialen Wandel, Vol 2. Göttingen: Schwartz; 1973. 74 p.

[5] Fleßa S. Systemisches Krankenhausmanagement. Berlin, Boston: De Gruyter; 2018. 802 p. ger.

[6] Hölzle K, Gemünden HG, Albers S, Gassmann O (2011). Schlüsselpersonen der Innovation. Handbuch Technologie- und Innovationsmanagement.

[7] Bundesministerium für Arbeit und Soziales. Sozialgesetzbuch (SGB) Fünftes Buch (V) - Gesetzliche Krankenversicherung: SGB V [Internet] [cited 2019 Feb 28]. Available from: https://www.sozialgesetzbuch-sgb.de/sgbv/135a.html.

[8] Bundesministerium für Arbeit und Soziales. Gesetz zur Verhütung und Bekämpfung von Infektionskrankheiten beim Menschen (Infektionsschutzgesetz): IfSG [Internet] [cited 2019 Feb 28]. Available from: https://www.gesetze-im-internet.de/ifsg/__23.html.

[9] Steinmann KE, Lehnick D, Buettcher M, Schwendener-Scholl K, Daetwyler K, Fontana M, et al. Impact of empowering leadership on antimicrobial stewardship: a single center study in a neonatal and pediatric intensive care unit and a literature review. Front Pediatr 2018;6:294. 10.3389/fped.2018.00294. PubMed PMID: 30370263. 10.3389/fped.2018.00294 PMC6194187 30370263

[10] Edwards R, Charani E, Sevdalis N, Alexandrou B, Sibley E, Mullett D (2012). Optimisation of infection prevention and control in acute health care by use of behaviour change: a systematic review. Lancet Infect Dis.

[11] Goerig Tillmann, Dittmann Kathleen, Kramer Axel, Diedrich Stephan, Heidecke Claus-Dieter, Huebner Nils-Olaf (2018). Infection control perception and behavior: a question of sex and gender? Results of the AHOI feasibility study. Infection and Drug Resistance.

[12] Bundesministerium für Gesundheit. Krankenhausstrukturgesetz: KHSG [Internet]; 2016 [cited 2019 Feb 5]. Available from: https://www.bundesgesundheitsministerium.de/service/begriffe-von-a-z/k/khsg.html.

[13] Martin Philippe, Abou Chakra Claire Nour, Williams Victoria, Bush Kathryn, Dyck Myrna, Hirji Zahir, Kiss Alex, Larios Oscar E., McGeer Allison, Moore Christine, Weiss Karl, Simor Andrew E. (2018). Prevalence of antibiotic-resistant organisms in Canadian Hospitals. Comparison of point-prevalence survey results from 2010, 2012, and 2016. Infection Control & Hospital Epidemiology.

[14] Bode Lonneke G.M., Kluytmans Jan A.J.W., Wertheim Heiman F.L., Bogaers Diana, Vandenbroucke-Grauls Christina M.J.E., Roosendaal Robert, Troelstra Annet, Box Adrienne T.A., Voss Andreas, van der Tweel Ingeborg, van Belkum Alex, Verbrugh Henri A., Vos Margreet C. (2010). Preventing Surgical-Site Infections in Nasal Carriers ofStaphylococcus aureus. New England Journal of Medicine.

[15] Chen Antonia F., Wessel Charles B., Rao Nalini (2013). Staphylococcus aureus Screening and Decolonization in Orthopaedic Surgery and Reduction of Surgical Site Infections. Clinical Orthopaedics and Related Research®.

[16] Nationales Referenzzentrum für Surveillance von nosokomialen Infektionen (2018). KISS Krankenhaus-Infektions-Surveillance-System, Modul MRSA-KISS. Referenzdaten. Berechnungszeitraum Januar 2017 bis Dezember 2017.

[17] Hübner N-O, Hutzschenreuter L, Dittmann K, Fleßa S (2018). PRIME: Herausforderungen und Lösungen bei der Einführung eines prästationären MRE-Screenings. Umwelt-Hygiene-Arbeitsmed..

[18] Hutzschenreuter L, Flessa S, Dittmann K, Hübner N-O. Costs of outpatient and inpatient MRSA screening and treatment strategies for patients at elective hospital admission - a decision tree analysis. Antimicrob Resist Infect Control 2018;7:147. 10.1186/s13756-018-0442-x. PubMed PMID: 30519461. 10.1186/s13756-018-0442-x PMC6267031 30519461

[19] Bader Lutz (2018). MRSA-Screening und -Dekolonisierung – Vergütungsregelungen im ambulanten Bereich: eine kritische Bilanz. Krankenhaushygiene up2date.

[20] GKV-Spitzenverband. Hygienesonderprogramm [Internet]; 2018. Available from: https://www.gkv-spitzenverband.de/krankenversicherung/krankenhaeuser/budgetverhandlungen/hygienesonderprogramm/kh_hygienesonderprogramm.jsp.

[21] GKV-Spitzenverband. Bericht des GKV-Spitzenverbandes zum Hygienesonderprogramm in den Förderjahren 2013 bis 2017. Berlin; 2018.

[22] Granig Peter, Perusch Sandra (2012). Prozessuale Identifikation und Bewertung von Innovationsrisiken im Krankenhaus. Innovationsrisikomanagement im Krankenhaus.

[23] Hilgers S (2011). DRG-Vergütung in deutschen Krankenhäusern.

[24] Deutsches Krankenhausinstitut. Anteil deutscher Krankenhäuser, die im Jahr 2016 einen Jahresüberschuss erwirtschafteten.: In Statista - Das Statistik-Portal [Internet] [cited 2019 Mar 13]. Available from: https://de.statista.com/statistik/daten/studie/425608/umfrage/anteil-deutscher-krankenhaeuser-einen-jahresueberschuss-erwirtschafteten/.

[25] Döring N, Bortz J (2016). Forschungsmethoden und Evaluation in den Sozial- und Humanwissenschaften.

